# LncRNA-SLC6A9-5:2: a potent sensitizer in ^131^I-resistant papillary thyroid carcinoma with PARP-1 induction

**DOI:** 10.18632/oncotarget.14578

**Published:** 2017-01-10

**Authors:** Cheng Xiang, Mao-lin Zhang, Qun-zi Zhao, Qiu-ping Xie, Hai-chao Yan, Xing Yu, Ping Wang, Yong Wang

**Affiliations:** ^1^ Department of Thyroid Surgery, Second Affiliated Hospital of Medical College, Zhejiang University, Hangzhou, Zhejiang Province, China

**Keywords:** thyroid cancer, lncRNA, ^131^I, PARP-1, SLC6A9-5:2

## Abstract

Recent studies have indicated that long non-coding RNAs play crucial roles in numerous cancers, including thyroid cancer, while their function in the mechanism of thyroid cancer ^131^I resistance has not been elucidated to date. The present study identified a functional long non-coding RNA, SLC6A9-5:2, which was involved in the radioactive therapy resistance of thyroid cancer. We demonstrated that SLC6A9-5:2 was remarkably downregulated in ^131^I-resistant thyroid cancer cell lines and ^131^I-insensitive patients and was positively correlated with Poly (ADP-ribose) polymerase 1 (PARP-1) expression and its activation. After downregulating SLC6A9 or blocking PARP-1 artificially, the sensitive thyroid cancer cells mostly displayed a tolerant phenotype under ^131^I exposure. Furthermore, SLC6A9-5:2 overexpression was positively correlated with PARP-1 mRNA and protein levels, which restored the sensitivity of resistant thyroid cancer cells. The present study further revealed that cancer cell death was primarily caused by ATP exhaustion in excessive DNA repair with high PARP-1 activity. In patients with thyroid cancer, a positive correlation between SLC6A9-5:2 and PARP-1 was identified, and low SLC6A9-5:2 expression was associated with a worse prognosis of papillary thyroid carcinoma. Hence, our data provide a new lncRNA-mediated regulatory mechanism implying that SLC6A9-5:2 can be used as a novel therapeutic target for ^131^I-resistant thyroid cancer.

## INTRODUCTION

Thyroid cancer ranks as the most common endocrine cancer and has the most rapidly increasing incidence rate of malignant cancer over the last several decades [1, 2]. After total or near total thyroidectomy in local invasion and distant metastasis cases, radioiodine (^131^I) is advocated with the aim of destroying occult microscopic foci of neoplastic cells within the remnant tissue or elsewhere and improving the long-term outcome in high-risk patients [3]. A major reason for these patients’ poor outcome is their failure to respond to radioiodine ablation therapy after surgical thyroidectomy due to the loss of radioiodine aggregate ability of thyroid follicular cells. Within this group, poorly differentiated carcinoma indicates higher rates of metastases and recurrence and is resistant to radioactive iodine (RAI) [4]. Although the overall survival rate of papillary thyroid carcinoma (PTC) is 97.7% at 5 years, in patients with postoperative ^131^I treatment, the recurrence rate was still reported to be as high as 15.6% in 3 years [5]. Once locoregional recurrence or distant metastases occur, many of these patients will never be cured with radioactive iodine therapy and will become RAI refractory with a 3-year overall survival rate of less than 50% [6]. Currently, novel molecular targeted agents and combination therapy are currently changing the natural history of RAI-refractory thyroid cancer treatment. The V600E BRAF mutant was shown to influence thyroid iodide-metabolism and decrease the absorptivity of ^131^I through the BRAF/MEK/MAP kinase pathway and may be an effective therapeutic strategy to treat PTC [7]. ^131^I treatment strategies have already been included in guidelines, while successful radioactive iodine (^131^I) remnant ablation is far from being understood [2]. Hence, we mainly focused on the discovery of more molecular biology features in^131^I resistance and try to identify new therapeutic targets in RAI-refractory patients.

Long non-coding RNAs (lncRNAs) are a class of non-coding RNAs containing over 200 nucleotides [8], largely accounting for the transcripts in the human genome and even protein-coding RNAs [9, 10]. In some cases, lncRNAs can serve as “molecular sink” transcription signal molecules for RNA-binding proteins. RNA: DNA binding elements also regulate heteroduplex formation in the chromosome and relevant molecular components as scaffold elements [11]. In the latest decade, accumulative evidence has implicated the role of lncRNAs in tumor proliferation and metastasis [12, 13]. For instance, the long non-coding RNA GAPLINC may stimulate SNAI2 and act as a transcription vector binding to PSF and NONO to promote colorectal cancer invasion [14]. In thyroid cancer, Kim identified 56 lncRNAs as potentially thyroid cancer-related genes, and demonstrated that lymph node metastasis of thyroid cancer and BRAF V600E mutation was closely related to LOC100507661 [15]. However, the roles of lncRNAs in the ^131^I sensitivity of thyroid cancer need further study. In the current study, the microarray for lncRNA discovered that lncRNA NONHSAT002850, identified as lnc-SLC6A9-5:2 (SLC6A9) in the Lncipedia database (www.lncipedia.org), was significantly decreased in ^131^I-resistant PTC cell lines compared with that in the sensitive cell line. This lncRNA variation was first described, and such variation was proven to play pivotal roles in thyroid cell ^131^I refractory.

During radioactive therapy,^131^I-enriched thyroid cancer cells suffer from radio-induced DNA damage with single- or double-strand breaks [16]. Poly (ADP-ribose) polymerase 1 (PARP-1), also known as a DNA damage sensor, binds to damaged DNA, thereby activating polymers of ADP-ribose (PAR) as the reaction of the cellular response to DNA strand breaks[17–20]. Until now, most of the studies have reported that PARP-1 plays a role as a DNA damage guardian and influences the radio sensitivity. Yao reported that the PARP-1 inhibitor 3-AB increased cigarette smoke-induced lung DNA damage [21]. PARP-1 could also be inhibited by miRNA with mRNA interference, which may serve as a new potential therapeutic targeting radiotherapy resistance in small cell lung cancer [22]. PARP-1 activation could maintain NF-κB activity, which is responsible for radio-sensitization, and the PARP-1 inhibitor AG-014699 could reverse NF-κB signal activation and mediate therapeutic resistance [23]. However, some other studies have shown that Parp-1 inhibition could protect cells from injury, and cell apoptosis was attenuated in under oxidative stress, similar to the radioactive conditions in bladder tissue [24]. However, the exact role of PARP-1 in thyroid cancer ^131^I sensitivity has not yet been elucidated. Therefore, our present study was focused on the relationship between ^131^I sensitivity in thyroid cancer cell lines with PARP-1 activity.

In this study, we analyzed the correlation between SLC6A9-PARP-1 pathway activity and ^131^I resistance. The dual-luciferase reporter assay was applied to verify that SLC6A9 could competitively bind to the PARP-1 promoter sequence. DNA damage sensor molecules such as PAR, H2AX and γH2AX, which are the activated products of PARP-1 during the DNA repair procedure, were chosen to indicate DNA damage after radiotherapy. Next, we illustrated the mechanism underlying how SCL6A9 and PARP-1 influenced the tolerance of thyroid cancer cells under ^131^I exposure. High SLC6A9 expression upregulated PARP-1 and signified ^131^I therapy sensitivity with ATP exhaustion during the high-strength DNA repair procedure induced by PARP-1 activation. We next analyzed the relationship between PARP-1 signaling and clinical characteristics in postoperative patients receiving ^131^I.

In conclusion, we demonstrated an lncRNA induced model in RAI-refractory thyroid cancer. As described, SLC6A9 was identified to promote PARP-1 expression and subsequently lead to cancer cell apoptosis under excessive DNA repair and ^131^I exposure.

## RESULTS

### Thyroid cancer cells are resistant to ^131^I after long-term exposure

**Figure 1:**
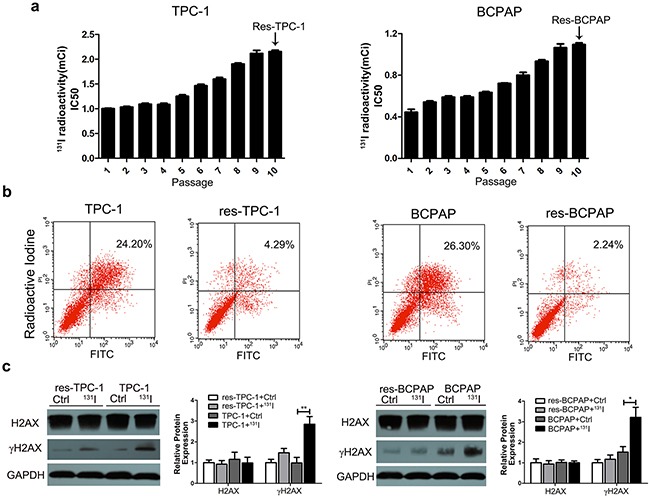
The long-term sub-lethal dose of ^131^I exposure induces the tolerance of thyroid cancer cells **a**. Treatment of the sub-lethal dose of ^131^I on continuously passaged TPC-1 and BCPAP thyroid cancer cell lines. ^131^I radioactivity was calculated with a half-time decay of 8.02 days, and the culture medium was changed every other day. ^131^I tolerance of cancer cells was significantly increased after 10 continuous passages. The 10th generation thyroid cancer cells were considered as ^131^I-resistant cells. **b**. Sensitive and resistant cell lines were treated with^131^I irradiation for 12 h, and apoptosis was measured by flow cytometry. **c**. Western blot analysis of the DNA damage marker γH2AX under ^131^I exposure. The data were from one representative experiment of three identically performed. The data were expressed as means±SD. * P<0.05;** P<0.01.

To acquire ^131^I-resistant thyroid cancer cell lines, wild-type PTC cell lines (^131^I-sensitive cell lines) TPC-1 and BCPAP were exposed to a sub-lethal ^131^I concentration. The ^131^I-resistant TPC-1 and BCPAP cells (res-TPC-1 and res-BCPAP) were obtained after continuous passaging for 10 generations. The median lethal intensity of ^131^I increased from 1.0 mCi/well to 2.2 mCi/well in the TPC-1 cell line and 0.5 mCi/well to 1.1 mCi/well in the BCPAP cell line (Figure 1a) compared with that in ^131^I-sensitive cells. We further identified that resistant cell lines could avoid apoptosis under ^131^I exposure as detected by flow cytometry (Figure 1b). After radioactive therapy, ^131^I treatment mainly led to cell death because of DNA damage, and γH2AX (the phosphorylated and activated form of H2AX, a DNA repair biomarker) was significantly expressed at a lower level in the resistant type of cells than in the control group, while H2AX expression remain unchanged (Figure 1c).

### SLC6A9 downregulation is accompanied by ^131^I resistance

**Figure 2:**
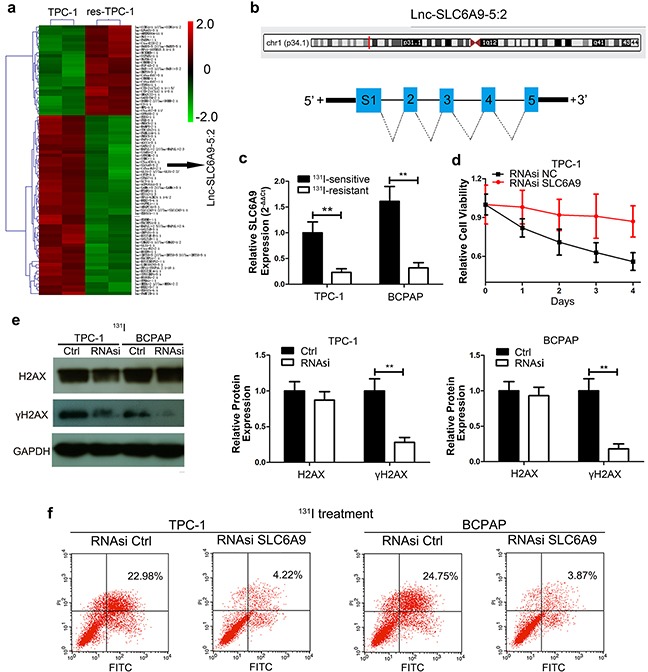
SLC6A9 was downregulated in resistant thyroid cancer cells and was correlated with ^131^I tolerance **a**. Hierarchical clustering analysis around the differentially expressed lncRNAs among ^131^I-sensitive and -resistant cells; each group includes two independently cultured TPC-1 cell lines. **b**. Schematic representation of the SLC6A9 location and five distinct fragments of SLC6A9-1. **c**. The SLC6A9 expression in sensitive and resistant thyroid cancer cells was analyzed by qRT-PCR. **d**. TPC-1 growth curves with SLC6A9 RNAsi or blank interference (50 nM) transfection with ^131^I treatment (n= 6 wells per group). The cell absorbance was measured every day for 4 continuous days. **e**. Western blot analysis of γH2AX after SLC6A9 RNAsi transfection for 48h followed by ^131^I treatment for 12h. **f**. The apoptosis rate percentage of TCP-1 and BCPAP cells of SLC6A9 RNAsi-transfected and control groups as measured by flow cytometry. The data are from one representative experiment of three identically performed. The data are expressed as means±SD. ** P<0.01.

To discover molecules that are responsible for the ^131^I-resistant phenotype, the hierarchical clustering analysis of microarray data showed over 70,000 lncRNAs alteration between TPC-1 and res-TPC-1 cell lines. Among them, SCL6A9 was one of the most significantly decreased lncRNAs in res-TPC-1 compared with that in TPC-1 cells (Figure 2a) and was confirmed to have a relationship with^131^I resistance subsequently. The location and structure of SLC6A9 are shown below (Figure 2b). Next, the expression of SLC6A9 was further validated to be expressed at a low level by qRT-PCR in PTC cells (Figure 2c). Thereafter, we knocked down SLC6A9 with RNAsi (Fig. S1a) to mimic the low SLC6A9 condition in the resistant cell type, significantly increasing the cell tolerance under ^131^I exposure (Figure 2d) (Supplementary Figure S1b). Additionally, we confirmed that the DNA repair intensity was less active after SLC6A9 interference compared with that in the RNAsi NC group (Figure 2e) and protected cancer cells from apoptosis with ^131^I treatment (Figure 2f). Hence, these data suggested that excess SLC6A9 content remarkably maintained ^131^I sensitivity.

### Overexpression of SLC6A9 enhances PTC cell sensitivity to ^131^I treatment

**Figure 3:**
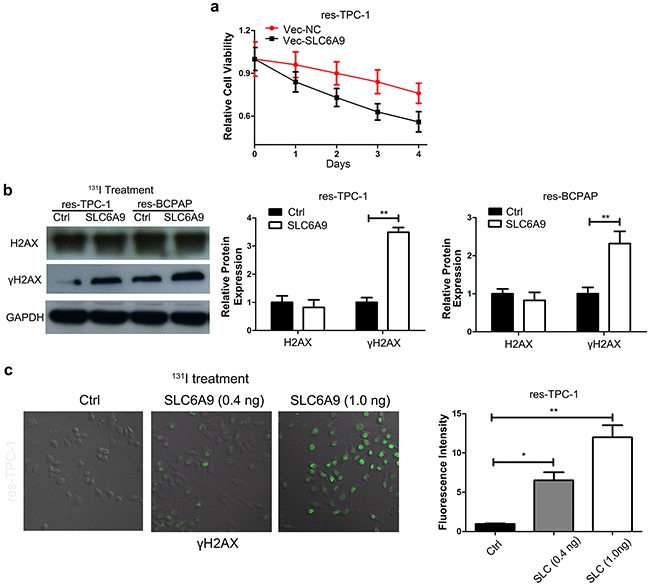
SLC6A9 overexpression leads to thyroid cancer cell sensitivity to ^131^I treatment **a**. TPC-1 growth curves with SLC6A9 plasmid or blank transfection following ^131^I treatment, (n= 6 wells per group). The cell absorbance was measured every day for four continuous days. **b**. Thyroid cancer cell DNA damage marker γH2AX expression after SLC6A9 plasmid transfection by Western blotting. **c**. Immunofluorescence images showing γH2AX expression (green) in situ after SLC6A9 plasmid transfection under^131^I treatment with the quantification displayed on the right. The data are from one representative experiment of three identically performed. The data were expressed as means±SD. *P<0.05;** P<0.01.

To further identify the role of SLC6A9 in the ^131^I tolerance characteristics of cancer cells, SLC6A9 was transferred into ^131^I-resistant TPC-1 and BCPAP cells (Supplementary Figure S2a). The transfection significantly reduces the survivability of the PTC cell lines under ^131^I treatment compared with that of the control group (Figure 3a) (Supplementary Figure S2b). On the other hand, γH2AX expression was also increased via transfection of SLC6A9 into ^131^I-resistant cell lines, which was confirmed by Western blot analysis (Figure 3b) and confocal laser scanning imaging (Figure 3c)(Supplementary Figure S2c) and indicated more serious cytotoxicity and DNA repair.

### SLC6A9 is positively correlated with PARP-1 expression, and PARP-1 inhibition makes thyroid cancer cells resistant to ^131^I

**Figure 4:**
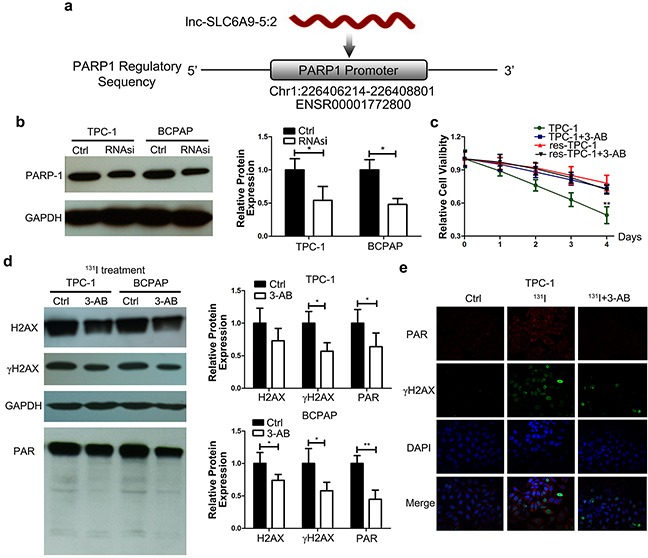
SLC6A9 is positively correlated with PARP-1 expression, and PARP-1 inhibition restored the ^131^I tolerance of thyroid cancer cells **a**. Illustration of the predicted target region of the PARP-1 promoter sequence with SLC6A9. **b**. PARP-1 protein was downregulated after transfection with SLC6A9 RNAsi (50nM). **c**. Survival curve of sensitive and resistant TPC-1 cells for 4 consecutive days of observation with PARP-1 inhibitor 3-AB treatment under ^131^I exposure by the MTT assay. 3-AB treatment made sensitive thyroid cancer resistant to ^131^I, while no difference was observed in the resistant group. **d**. DNA repair intensity-related protein PAR and γH2AX expression under ^131^I exposure with 3-AB treatment for 24 h. **e**. DNA repair intensity in the Ctrl, ^131^I and ^131^I+3-AB groups with double-labeled immunofluorescence of PAR and γH2AX. The nuclei were stained with DAPI. 3-AB significantly weakened DNA repair under ^131^I treatment. Co-localization is demonstrated in the merged image. Magnification:×400. The data were expressed as means±SD. *P<0.05;**P<0.01.

To reveal the underlying mechanism of SLC6A9 downregulation in resistant cancer cells, a continuous complementary region was identified in the PARP-1 promoter sequence when we inspected the genomic sequence of SLC6A9 (Figure 4a). Additionally, the PARP-1 gene plays an important role during DNA repair, which we infer to respond to radioactivity-induced DNA damage. We introduced SLC6A9 siRNA into cancer cells, and PARP-1 expression was significantly decreased (Figure 4b). Next, we transfected SLC6A9 encapsulated plasmid into cancer cells, and PARP-1 was found to be positively correlated with SLC6A9 expression (Supplementary Figure S3a). To further explore the role of PARP-1 during ^131^I exposure, 3-AB (a PARP-1 inhibitor) was introduced to inhibit PARP-1 activity (Supplementary Figure S3b) and block the SLC6A9-PARP-1 signaling pathway. As a result, sensitive thyroid cancer cells actually became resistant to ^131^I treatment after 3-AB treatment, and no influence was found in the res-TPC-1 cell line (Figure 4c). Such a phenomenon was also observed in the BCPAP and res-BCPAP cell lines (Supplementary Figure S3c), indicating that PARP-1 was indispensable for ^131^I sensitivity. Furthermore, 3-AB was shown to weaken cancer cell DNA repair by Western blot analysis of PAR (active product of PARP-1) and γH2AX (Figure 4d), as well as by confocal laser scanning imaging (Figure 4e) (Supplementary Figure S4d). The inhibition of PARP-1 activity might be indicative of cancer cell survival with decreased DNA repair. Additionally, SLC6A9 downregulation in resistant cancer cells interfered with PARP-1 activity to influence therapeutic efficacy.

### Fragment 1 of SLC6A9 is the functional segment to promote PARP-1 expression

**Figure 5:**
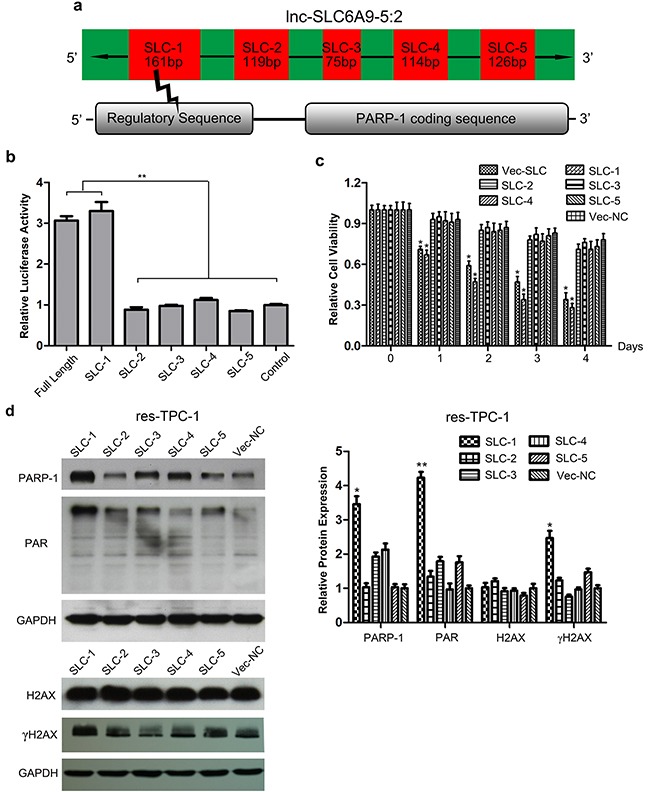
Fragment 1 of SLC6A9 (SLC6A9-1) regulates PARP-1 expression and activity **a**. Illustration of the SLC6A9 structure and target region where SLC6A9 binds to the PARP-1 promoter. **b**. Luciferase activity assays for full-length SLC6A9 and its five segments within the PARP-1 promoter region. The wild-type SLC6A9 sequence was co-transfected with the predicted target sequence, respectively. Both SLC6A9-1 and full-length SLC6A9 enhanced the luciferase activity of the targeted reporter. There was no obvious difference in the luciferase activity for SLC6A9-2, -3, -4, and -5, and the randomly built negative control sequence reporter. The graphs represent data from 3 separate experiments and five identical wells. **c**. The overexpression of SLC6A9-1 sensitized thyroid cancer cells to ^131^I treatment (n=6). **d**. Western blot analysis indicated that the upregulation of SLC6A9-1 resulted in a significant increase of the DNA-repair associated proteins PARP-1, PAR and γH2AX with ^131^I treatment. The data were expressed as means±SD. *P<0.05;**P<0.01.

As previously described, SLC6A9 constitutes 5 separated segments during transcription. It is predicted that the first fragment of SLC6A9 (SLC6A9-1) was the functional segment that combined with the regulatory sequence of PARP-1 (Figure 5a). To verify that SLC6A9 directly targeted the PARP-1 promoter sequence, the PARP-1 promoter seed sequence recognition site was cloned into a Dual-Luciferase reporter. The results showed that the relative luciferase activity of the plasmid carrying PARP-1-UTR-WT was significantly increased in the presence of SLC6A9 or SCL6A9-1 (Figure 5b). In accordance with our expectation, in the resistant cell type, transfection of SLC6A9 or SLC6A9-1 could induce their sensitivity to ^131^I cytotoxicity, while SLC6A9-2, -3, -4, 5 or blank vector transfection made no difference (Figure 5c)(Supplementary Figure S4a). Furthermore, transfection of SLC6A9-1 into resistant thyroid cancer cells also enhanced the expression of PARP-1, PAR and γH2AX, indicating more active DNA repair (Figure 5d)(Supplementary Figure S4b). Therefore, it could be inferred that SLC6A9-1 directly bound to the PARP-1 promoter sequence and acted as the functional region of SLC6A9.

### SLC6A9/PARP-1 activation leads to ATP/ADP exhaustion under ^131^I exposure

**Figure 6:**
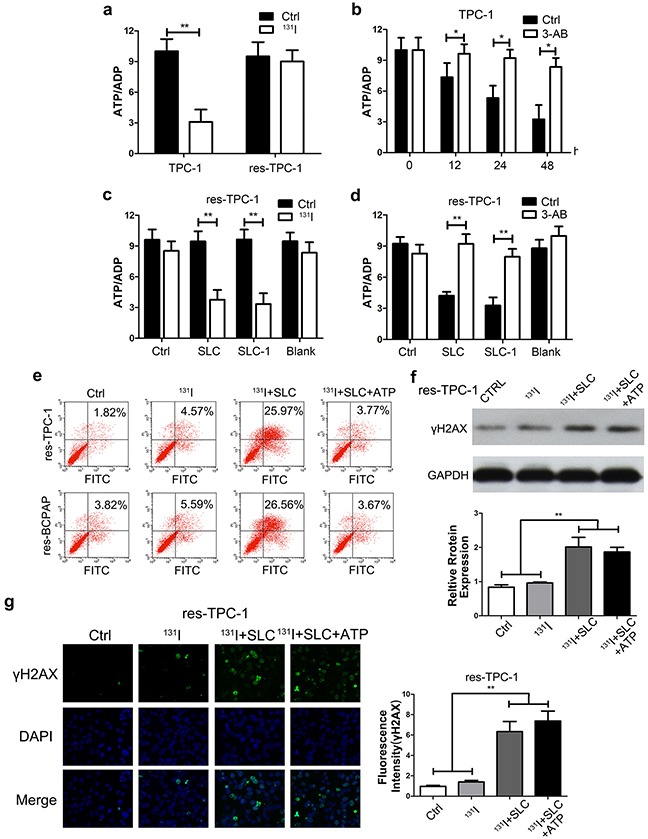
Upregulation of the SLC6A9-PARP-1 pathway enhanced the sensitivity to ^131^I treatment through energy exhaustion during excess DNA repair **a**. The ATP/ADP ratio in TPC-1 cells under ^131^I exposure. The ATP and ADP content was calculated according to the standard curve previously drawn (n=3). **b**. The ATP/ADP ratio in the TPC-1 cell line treated with 3-AB and ^131^I. The energy consuming condition was detected every 12h using a detection kit. **c**. The ATP/ADP ratio with SLC6A9 and SLC6A9-1 transfection, which protected thyroid cancer cells from energy exhaustion. **d**. 3-AB reversed the energy exhaustion condition induced by SLC6A9 and SLC6A9-1 in res-TPC-1 cells. **e**. Supplementation with ATP (2mM) protected thyroid cancer cells from apoptosis in SLC6A9-transfected thyroid cancer cells. **f, g**. DNA repair was demonstrated in the SLC6A9 and SLC6AP+ATP groups with γH2AX observation. In the SLC6A9 and SLC6AP+ATP groups, DNA repair both remained at a high level and showed no difference. The data were expressed as means±SD. *P<0.05; **P<0.01.

Next, we investigated why high DNA repair intensity led to low ^131^I tolerance in high SLC6A9 cells. First, we found that, in wild-type cells, the ATP/ADP ratio dropped dramatically after ^131^I exposure. However, the ATP/ADP ratio remained constant in the resistant group (Figure 6a)(Supplementary Figure S5a). These results suggested that resistant cancer cells could restore their ATP content through the inhibition of DNA repair. Furthermore, we pretreated PTC cells with the PARP-1 inhibitor 3-AB for 12h, 24h and 48h before ^131^I treatment, significantly protecting thyroid cancer cells from ATP exhaustion (Figure 6b)(Supplementary Figure S5b). Importantly, SLC6A9 and SCL6A9-1 transfection, to a large extent, led to energy exhaustion in resistant cells with ^131^I treatment (Figure 6c)(Supplementary Figure S5c). 3-AB treatment could defuse the sensitizing effect of SLC6A9 or SLC6A9-1 transfection (Figure 6d) (Supplementary Figure S5d). In further research, we introduced exogenous ATP into the cultures to overcome the energy crisis. It was observed that ATP supplementation protected cancer cells from apoptosis in SLC6A9-transfected sensitive cancer cells (Figure 6e). However, ATP supplementation did not influence the DNA repair intensity in high SLC6A9-expressed cells using Western blotting and immunofluorescence of γH2AX (Figure 6f, 6g) (Supplementary Figure S5e). The above results indicated that, following^131^I treatment, the SLC6A9 content could maintain PARP-1 activation to fight against DNA damage, and this process led to ATP exhaustion to enhance thyroid cancer cell sensitivity.

### SLC6A9 expression is positively correlated with PARP-1 expression in tissue specimens, and low SLC6A9 expression indicates a worse prognosis clinically

**Figure 7:**
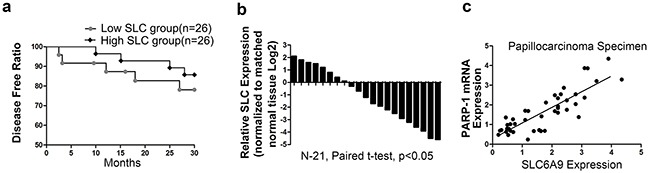
Low SLC6A9 expression predicted a worse prognosis in ^131^I-treated patients **a**. In a group of 52 patients receiving ^131^I treatment, qRT-PCR showed that high SLC6A9 had a higher disease-free rate compared with the low SLC6A9 group by Kaplan-Meier curve analysis. **b**. SLC6A9 expression was measured by qRT-PCR in thyroid cancer tissues and adjacent normal thyroid tissues. The results were expressed as relative Log^2^ ratios. **c**. The relationship between PARP-1 and SLC6A9 expression was explored by Spearman's correlation in thyroid cancer specimens (r=0.715).

Fifty-two patients were followed up to 30 months. It was discovered that the disease-free rate was significantly lower in patients with low SLC6A9 expression (Figure 7a). Among 21 randomly assigned patients with ^131^I-resistant cancer, it was discovered that SLC6A9 expression was much lower in cancer cell tissues than in adjacent normal tissues (Figure 7b). Additionally, SLC6A9 was positively correlated with PARP-1 mRNA expression (Figure 7c). The results above indicated that PARP-1 and SLC6A9 had a close relationship clinically and might act as potential treatment targets for ^131^I-resistant PTC patients.

## DISCUSSION

The development of PTC ^131^I refractoriness involves various genetic and epigenetic mechanisms. In recent years, lncRNAs initially drew attention in cancer development such as melanoma, glioblastoma and esophageal cancer, as well as thyroid cancer [25, 26]. Our study was the first to demonstrate the molecular mechanism of lncRNAs in the ^131^I resistance of thyroid cancer. In the current study, we reported that a low level of SLC6A9 indicates ^131^I resistance of thyroid cancer cells. In addition, we found that lower SLC6A9 indicated higher recurrence possibility after ^131^I treatment. These findings strongly support the hypothesis that SLC6A9 is essential for the radio-sensitive maintenance of PTC.

It remains unknown the mechanism underlying how lncRNAs influence gene expression concerning radiosensitivity. Several recent studies have suggested that the regulatory mechanism encompassed almost every aspect of the pre- and post-transcriptional processes, including transcription, post-transcriptional processing, chromatin modification, and the modulation of protein function [27, 28]. For example, lnc-ATB could upregulate ZEB1 and ZNF-217, and then induce EMT, leading to trastuzumab resistance and invasion-metastasis in breast cancer [29]. LncRNA-MALAT1 binds topolycomb repressive complex 2 and suppresses PCDH10, and promotes gastric progression [30]. In this study, we identified SLC6A9 as a regulatory factor through targeting the PARP-1 promoter by sequence complementarity analysis. Here, we demonstrate that SLC6A9 was recruited to the PARP-1 promoter region and enhanced PARP-1 transcription. Higher SLC6A9 expression significantly increased PARP-1 expression in thyroid cancer cells and was accompanied by effective ^131^I treatment.

PARP-1 activation is highly correlated with the DNA damage response that is promoted by DNA repair and genomic integrity [17, 18]. Among the 18 members of the PARP enzyme family, PARP-1 accounts for more than 90% activity of the cells and could be activated by DNA breaks, additionally, NAD+ is cleaved into nicotinamide and ADP-ribose. Thereafter, long-branched PAR polymers are synthesized covalently attached to acceptor proteins. Under mild genotoxic stimulation, the activation of PARP-1 may facilitate DNA repair and maintain cell survival [31]. After ultraviolet lesion formation, PARP-1 could simultaneously bind to damaged DNA and participate in DNA repair [16]. The use of PARP-1 inhibitors was applied as radiosensitizers in prostate patients with localized ETS fusionpositivity [32]. As a result, we initially hypothesized that high PARP-1 activity implies ^131^I resistance because of timely DNA repair. However, in our study, PARP-1 inhibition dramatically protected the thyroid cancer cells from radioactive therapy to a great extent. In many oxidative stress pathophysiological conditions, over-activation of PARP was induced increased free radicals. PARP over-activation consumes most of the intracellular NAD+, causing ATP storage to fulfill the huge energy demand and finally leading to cell dysfunction [33, 34]. In some other studies, PARP activation was inhibited, and the low activation of PARPs was widely considered to help cells avoid apoptosis under stress [24, 35]. Thus, we further focused on how PARP-1 inhibition could protect thyroid cancer cells from ^131^I injury. We found that the ATP/ADP ratio was much higher in the PARP-1 inhibition group with less DNA repair. It has been reported that cell death was related to the extent of ATP depletion: a medium ATP decrease may cause cell apoptosis and a severe ATP decrease may lead to cell necrosis [36, 37]. Similarly, Yang found that PARP-1 inhibition with 3-AB may protect renal cells from apoptosis in hemodynamic disorders during an acute kidney injury mode through ATP restoration [35]. The above results implied that PARP-1 overactivation caused by ^131^I radioactivity was accompanied by ATP crisis, triggering cancer cell death because of energy depletion. Therefore, it is likely that maintaining PARP-1 activity may play an important role during ^131^I radioactive treatment in PTC. In our study, PARP-1 inhibition makes PTC more tolerant to ^131^I treatment. As a result, we aimed to explore a regulatory factor to increase PARP-1 activation and enhance ^131^I sensitivity. Based on our lncRNA expression microarray result, SLC6A9 was predicted to have a relationship with PARP-1 expression. Following SCL6A9 transfection, the ATP ratio was significantly decreased, and the ^131^I-resistant phenotype in the PTC was reversed to a large extent. In a recent study, artificial knockdown of high PARP-1 expression under oxidative stress was responsible for the protection of mitochondria to maintain cell survival [38]. Hence, we specifically introduced SCL6A9, which is a PARP-1 promoter to mimic the DNA repair stress condition under ^131^I treatment. These findings may be beneficial for maintaining the ^131^I sensitivity in the PTC treatment procedure.

Most of the time, lncRNAs were pieced with intermittent fragments during RNA translation and played roles through specific binding areas of the gene locus [39]. Based on database information, 5 adjacent fragments constitute the SLC6A9 sequence; within these, fragment 1 was predicted to have a complementary correlation with PARP-1 promoter bases. As a result, we found that only fragment 1 of SLC6A9 transfection could significantly transform ^131^I-resistant thyroid cancer cells into the sensitive phenotype. Furthermore, our analyses revealed high SLC6A9 and SLC6A9-1 expression promote PARP-1 function in thyroid cancer. Because the present study showed that SLC6A9 significantly maintained ^131^I sensitivity of thyroid cancer *in vitro*, our study did not investigate the systematic effects of SCL6A9 considering the target efficiency in PTC tumors that is an important point in the decision making process of molecular target treatment. We will try to establish an orthotropic model to further evaluate the effects of SLC6A9 on thyroid cancer *in vivo*.

In conclusion, this study demonstrated that SLC6A9 is downregulated in ^131^I-resistant thyroid cancer accompanied by PARP-1 inhibition. A high level of SLC6A9 is closely associated with an overall-disease free rate of PTC patients under ^131^I therapy. Moreover, the ectopic expression of SLC6A9 and knockdown of PARP-1 generally influence the effects of ^131^I treatment. Therefore, our results imply that SLC6A9 acts as a stabilizer for ^131^I sensitivity by maintaining PARP-1 expression and may serve as a novel therapeutic agent for thyroid cancer patients with ^131^I treatment failure.

## MATERIALS AND METHODS

### Patients and tissue samples

Tissue samples were collected from patients undergoing surgery at the Thyroid Surgery Department, the Second Affiliated Hospital (Hangzhou, China) from January 2014 to January 2015. During the period, 52 patients diagnosed with papillary thyroid carcinoma with local metastasis from ages 23 to 64 years were collected. The patients were treated with total thyroidectomy and ^131^I radiotherapy following the 2009 American Thyroid Association Management Guidelines [40]. Additionally, the surrounding para-carcinoma tissue samples were collected and were verified as normal thyroid tissue with a pathological diagnosis. Institutional Ethics Review Board approval and informed consent were obtained prior to the initiation of the study. The samples were stored at −80°C immediately after collection.

### Cell culture

Cells were incubated in 5% CO_2_ at 37°C and were cultured in complete medium composed of 90% RPMI-1640 (Gibco) and 10% fetal bovine serum (Gibco), supplemented with 100 U/ml penicillin and 100 mg/ml streptomycin (HyClone). Thyroid cancer cell lines, TPC-1 and BCPAP, were bought from American Type Culture Collection (ATCC) in July 2015. Cell lines were authenticated for the genotypes by DNA fingerprinting and was passaged for no more than 6 months following resuscitation. To build the ^131^I-resistant thyroid cancer model, the cells were cultured in 6-well plates under continuous exposure of sub-lethal ^131^I.

### RNAsi and plasmid transfection

The SLC6A9 interference sequence was designed and synthesized by Invitrogen Co. (Shanghai, China). The cells were seeded into plates and were incubated overnight with 30% coverage, and the cells were transfected with SLC6A9 RNAsi or the matched negative controls (NC) (Ribobio, Guangzhou, China). Transfection was conducted using Lipofectamine 3000 (Invitrogen) according to the manufacturer's instructions. The same method was also used for the SLC6A9 plasmid and matched NC vector transfection. After 48h of tranfection, the cancer cells were collected for further RNA/protein extraction.

### Quantitative real-time polymerase chain reaction (qRT-PCR)

Total RNA was extracted from either tissue samples or cells with Trizol reagent (Takara) according to the manufacturer's instructions. qRT-PCR was applied to evaluate the expression of lncRNA and mRNA using the SYBR Green PCR Kit (Takara) in the Applied Biosystems StepOne-Plus Real-Time PCR System. The RNA levels were calculated according to the 2^−ΔΔCt^ system compared with the NC primer group. SLC6A9 was identified as being highly expressed if the relative RNA level was above the median value. All of the reactions were performed in triplicate.

### Western blot analysis

Cell lysates were extracted from reagent-interfered cells using the radioimmunoprecipitation assay (RIPA) with 1% proteinase inhibitors. Protein extracts were separated by 10% sodium dodecyl sulfate-polyacrylamide gel electrophoresis (SDS-PAGE). Next, the separated proteins were transferred onto PVDF membranes (Millipore, UK). The membranes were then blocked and incubated with primary antibodies overnight at 4°C. The primary antibodies included PARP-1 (Catalog number: 13371-1-AP, Proteintech), PAR (Catalog number: ab14459, Abcam), H2AX (Catalog number: #7631, Cell Signaling Technology), and γH2AX (Catalog number: #5438, Cell Signaling Technology). After washing with TBST, the membranes were incubated with secondary antibodies (Invitrogen), and the proteins were visualized by electrochemiluminescence. Finally, the band brightness density was evaluated by Bio-Rad Quantity One software.

### Cell proliferation assay

In total, 3×10^3^ cells were counted and seeded in 96-well plates and were cultured at 37°C. After transfection or other management, 20 μl of MTT solution per well was added, and the plates were continued to incubate for another 4 h at 37°C. Next, the medium was replaced by 150 μl of DMSO (Sigma) in each well. After 15 min of dissolving, the absorbance was measured at 570 nm using a microplate reader (Sunrise, Tecan). The MTT assay was repeated three times in five identical replicates.

### LncRNA microarray assay

The LncRNA microarray chip contained more than 70,000 identified lncRNA sequences. Affymetrix OElnc was purchased from Oebiotech Company (Shanghai, China). First, these RNA samples were qualitatively identified up to standard with the Agilent Bioanalyzer 2100 (Agilent Technologies). Next, the samples were cleaned and hybridized in accordance with the Agilent Gene Expression Analysis protocol. Original data were obtained by the Affymetrix Gene Chip Command Console version 4.0 (Affymetrix) and was further analyzed by Expression Console software (Affymetrix).

### Luciferase reporter assay

To confirm whether SLC6A9 could directly bind to the PARP-1 regulatory region, the plnc-Reporter and control were structured and purchased from RIBOBIO (Guangzhou, China). Briefly, the thyroid cancer cell lines were seeded in 96-well plates (30–40% confluence) and were transfected with the luciferase vector (100 nM) with or without the pre-built promoter sequence by Lipofectamine 3000 (Invitrogen). After 48h of incubation, luciferase activity was determined using the dual luciferase assay system (Promega). The relative luciferase activity was normalized to control firefly luciferase activity.

### ATP and ADP measurements

Measurements of the ATP and ADP substances were performed according to a previous study [35]. As a result, the absorbance spectrum of ATP and ADP coloration was examined at 254nm. The concentrations of these energy substances were calculated compared with the standard curve, which was prepared in the previous study.

### Measurement of the cell apoptosis rate

The thyroid cancer cell apoptosis rate was determined by flow cytometry. Briefly, cells were harvested and washed twice with mild phosphate-buffered saline (PBS), which was further mixed with binding buffer at a density of 2×10^6^/ml. Next, thyroid cancer cells were fixed with Annexin V-FITC according to the manufacturer's protocol. The signal intensity was acquired by an FACS Calibur flow cytometer (BD Biosciences) and was analyzed with related software. Each tube was measured in triplicate as independent experiments.

### Immunofluorescence and confocal laser scanning microscopy (CLSM)

Thyroid cancer cells on glass slides were fixed in 4% paraformaldehyde at room temperature for 15 min. After blocking with serum in pre-cooled phosphate-buffered saline, the slides were incubated with γH2AX, PARP-1 or PAR antibodies at 4°C overnight. Next, the samples were washed and incubated with a secondary antibody (Jackson ImmunoResearch Lab Inc) for 2h. After rinsing with PBS, the cells were stained with DAPI (BD Bioscience). Images were captured with a confocal laser scanning microscope (Carl Zeiss).

### Statistical analysis

All statistical analyses were performed using SPSS13.0 statistical software. All of the values were presented as the means ± SD. Differences between groups were assessed using unpaired t-test. SLC6A9 expression and clinical characteristics were analyzed by χ^2^ test, while the correlation between SLC6A9 and protein expression was calculated with Spearman's correlation. P< 0.05 was considered to be statistically significant.

## SUPPLEMENTARY FIGURES


