# Supplementary material for: LncRNA-SLC6A9-5:2: a potent sensitizer in 131I-resistant papillary thyroid carcinoma with PARP-1 induction
Source: Oncotarget. 2017 Jan 10;8(14):22954–67. doi: 10.18632/oncotarget.14578 (PMC5410277; doi:10.18632/oncotarget.14578)
Supplement: Supplementary file 1 [file oncotarget-08-22954-s001.pdf]

LncRNA-SLC6A9-5:2: a potent sensitizer in ^{131}I-resistant papillary thyroid carcinoma with PARP-1 induction

SUPPLEMENTARY FIGURES

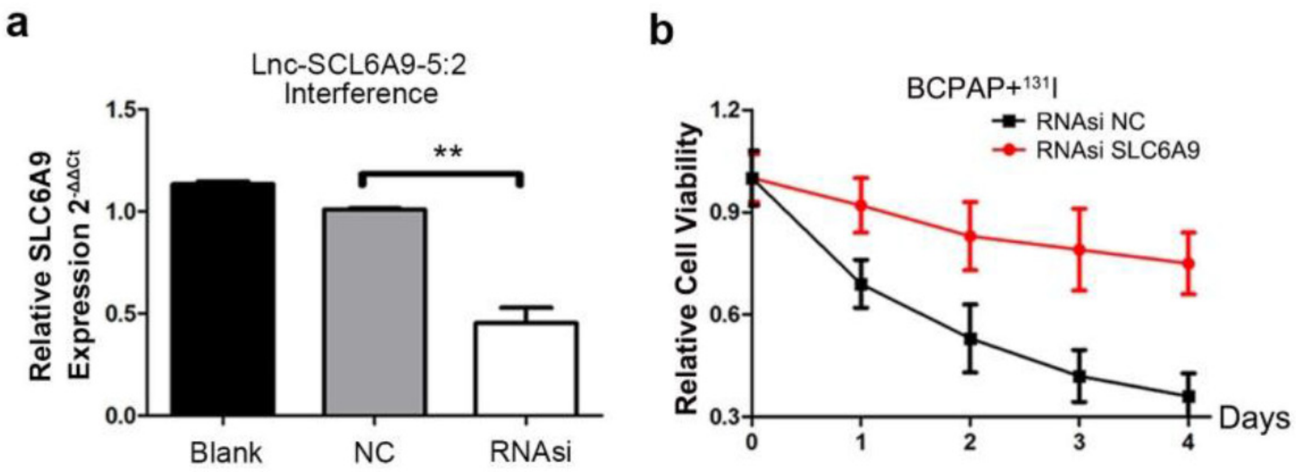

Supplementary Figure S1: SLC6A9 influenced PTC 131I tolerance. a. SLC6A9 expression with SLC6A9 siRNA (50 nM) transfection. **b.** The growth curve of BCPAP cells with SLC6A9 interference (50 nM) under 131I treatment.

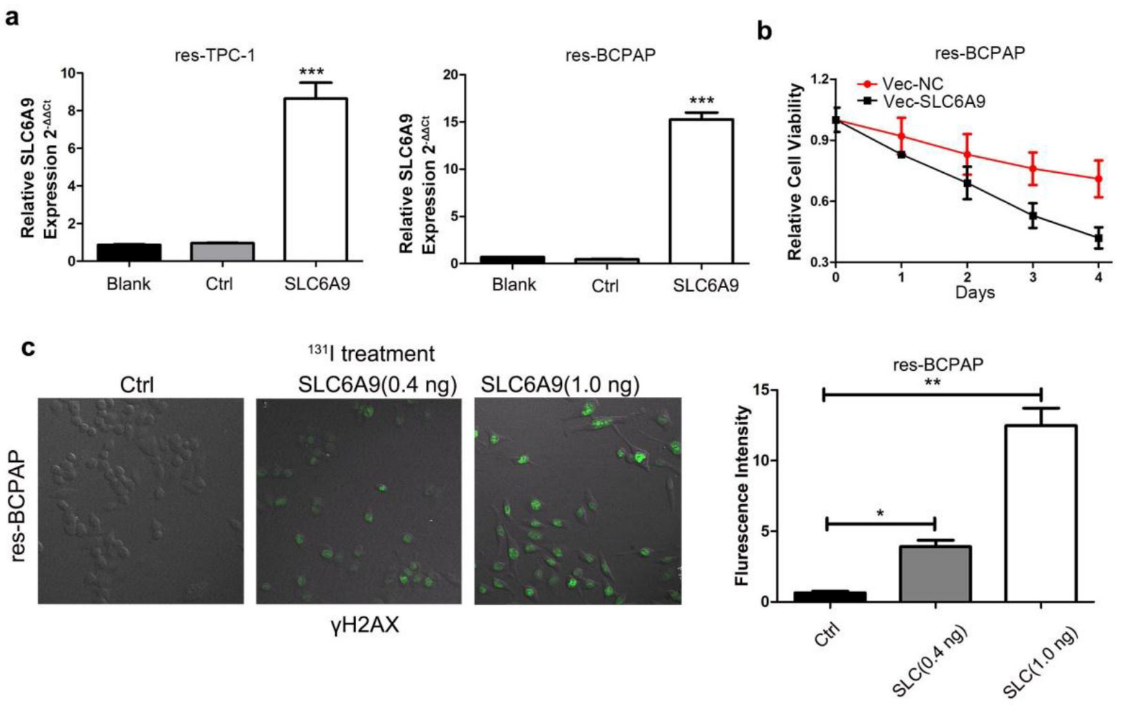

Supplementary Figure S2: SLC6A9 over expression caused thyroid cancer cell sensitivity to 131I. a. SLC6A9 expression with SLC6A9 plasmid transfection in thyroid cancer cells. **b.** Growth curve of res-BCPAP with SLC6A9 overexpression in 131I treatment. **c.** DNA repair intensity with γH2AX fluorescence observation after SLC6A9 transfection (0.4 ng and 1.0 ng/well).

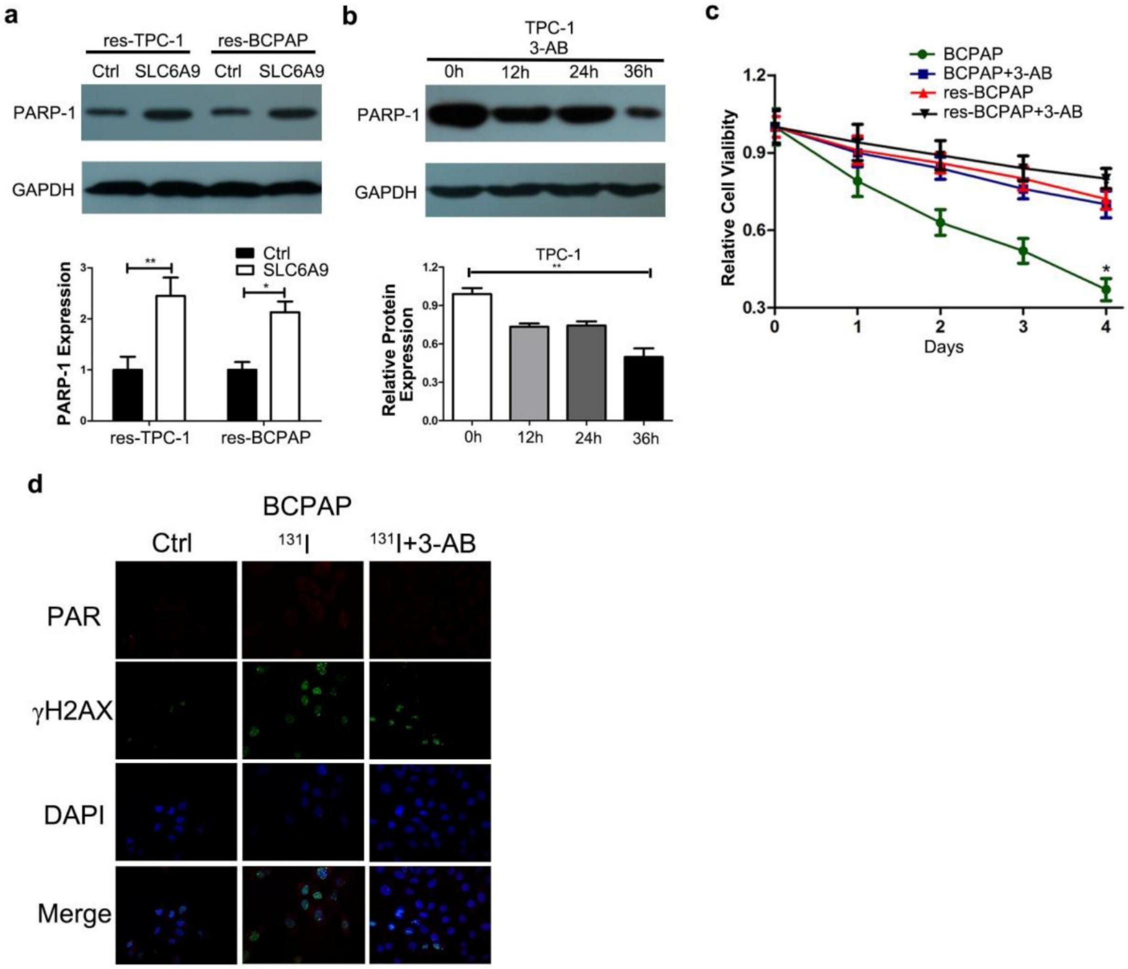

Supplementary Figure S3: Correlation between SLC6A9 and PARP-1 expression and their influence on 131I sensitivity. **a.** PARP-1 expression after SLC6A9 plasmid transfection in resistant PTC cells. **b.** PARP-1 expression after 3-AB treatment for 12h, 24h and 36h. **c.** Effect of the PARP-1 inhibitor 3-AB on the BCPAP survival curve in 131I treatment with the MTT assay. **d.** Expression of DNA repair-related proteins PAR and γH2AX with fluorescence observation after 24h of 3-AB treatment.

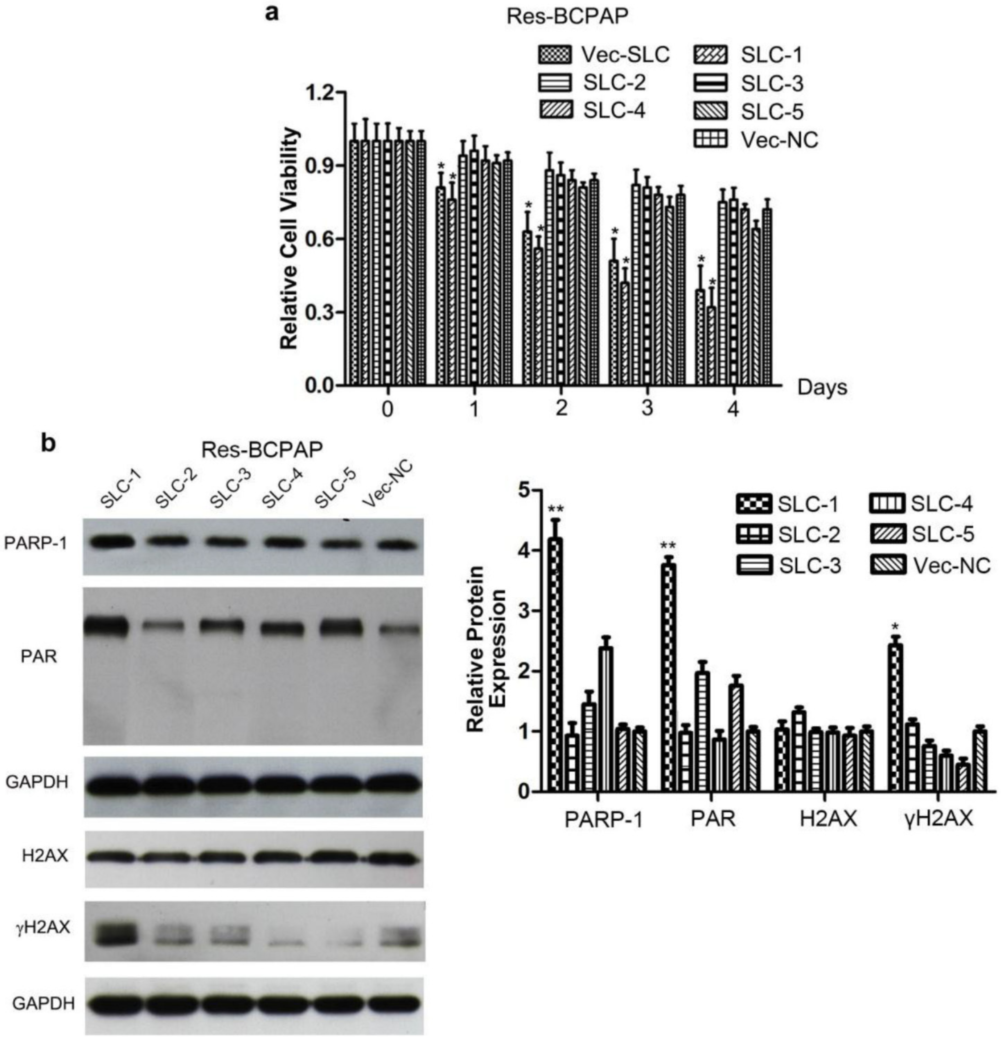

Supplementary Figure S4: SLC6A9-1 leads to PTC cell death after 131I treatment. a. Growth curve of BCPAP with SLC6A9 and SLC6A9-1-5 vector transfection during 131I treatment. **b.** Western blot analysis indicated that the upregulation of SLC6A9-1 resulted in a significant increase in PARP-1, PAR and γH2AX in the 131I treatment of the BCPAP cancer cell line. *P<0.05, **P<0.01.

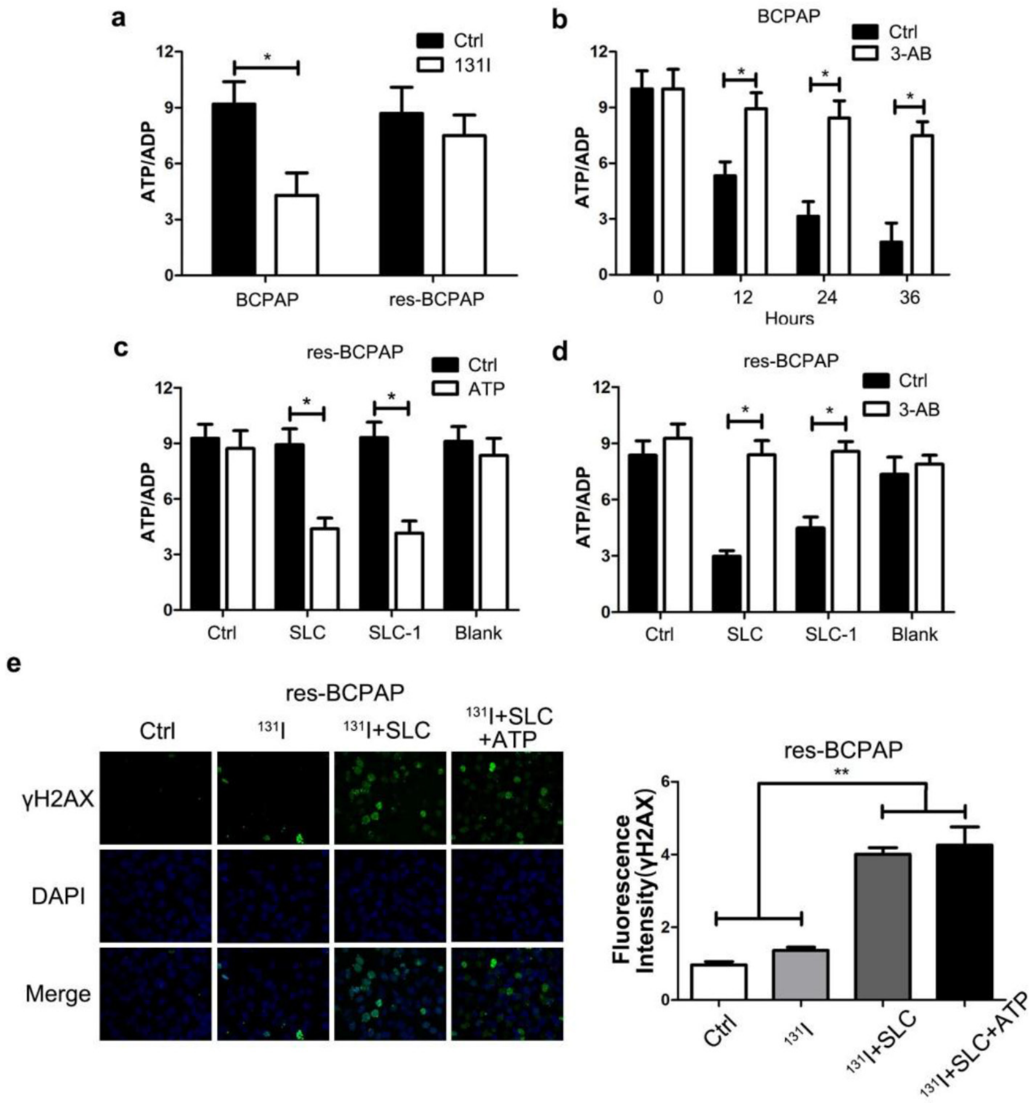

Supplementary Figure S5: SLC6A9 caused thyroid cancer sensitivity to 131I treatment with energy exhaustion. a-d. The ATP/ADP ratio decreased after 131I treatment while SLC6A9-PARP-1 activation accelerated the energy exhaustion in the BCPAP cell line. **e.** DNA repair intensity with γH2AX staining in the BCPAP cell line transfected with SLC6A9 or supplemented with ATP.
